# Monitoring renal function during combination therapy with telaprevir in HIV/HCV co-infected patients with advanced/fibrosis cirrhosis

**DOI:** 10.7448/IAS.17.4.19627

**Published:** 2014-11-02

**Authors:** Cinzia Puzzolante, Marco Vecchia, Stefano Zona, Vanni Borghi, Giovanni Guaraldi, Cristina Mussini

**Affiliations:** Azienda Ospedaliero-Universitaria Policlinico di Modena, Divisione di Malattie Infettive, Modena, Italy

## Abstract

**Introduction:**

Telaprevir (TVR) plus peg interferon (PEG-IFN) and ribavirin (RBV) substantially increase treatment efficacy for genotype 1 chronic hepatitis C virus (HCV) infection but data about its safety in HIV patients with cirrhosis are lacking. Our purpose was to evaluate estimated Glomerular Filtration Rates (eGFR) variations during combination therapy in a difficult-to-treat HIV/HCV population with advanced fibrosis/cirrhosis through three different scores commonly used in clinical practice: CKD-EPI (Chronic Kidney Disease Epidemiology Collaboration), Modification of Diet in Renal Disease (MDRD) scores (that consider gender, ethnicity, age, serum creatinine level [SCL]) and MDRD 6 variable (that considers the previous parameters plus blood urea nitrogen and serum albumin). Second objective was to identify any association between creatinin clearance and haemoglobin (Hb) variation during combination therapy.

**Materials and Methods:**

We conducted an observational retrospective study including 18 HIV/HCV patients attending our clinic who started combination therapy for HCV, including in the analysis the first 16 weeks of therapy. Treatment included TVR 1125 mg BID (twice a day) for 12 weeks, PEG-IFN α-2a 180 mcg QW and RBV daily dose according to body weight (800 mg in individuals <60 kg; 1000 mg in individuals 60–75 kg; 1200 mg in individuals >75 kg). Advanced fibrosis was defined as Metavir score=F3 or Ishak 3–4 and cirrhosis was defined as Metavir score F4 or Ishak 5–6 assessed by liver biopsy or transient elastography respectively. P per trend were assessed to evaluate any change of SCL and eGFR (according to different formulas). Multilevel linear regression analysis was performed to estimate factors associated with reduction of Hb.

**Results:**

Baseline characteristics and HCV virological responses were collected. Figure 1 shows median SCL and eGFR trends across follow-up period: SCR p-per-trend was 0.28, for CKD-EPI was 0.50, for MDRD was 0.48, for MDRD-6 variables was 0.27. Hb trend was also assessed and a significant decrease of Hb across follow-up (p per trend <0.001) was noted. Multilevel regression analysis found an association between SCL and Hb change, after adjustment for age, sex, basal BMI, tenofovir exposure and proteinuria (assessed at baseline) as shown in Table 1.

**Conclusions:**

In our experience, combination therapy with TPV in cirrhotic HIV-HCV patients did not significantly affect renal function. As expected, Hb levels decreased during treatment and it is likely related to RBV exposure. In multivariable analysis, reduction of Hb appeared to be related to SCL higher levels, thus suggesting even a mild decrease of renal function.

**Figure 1:**
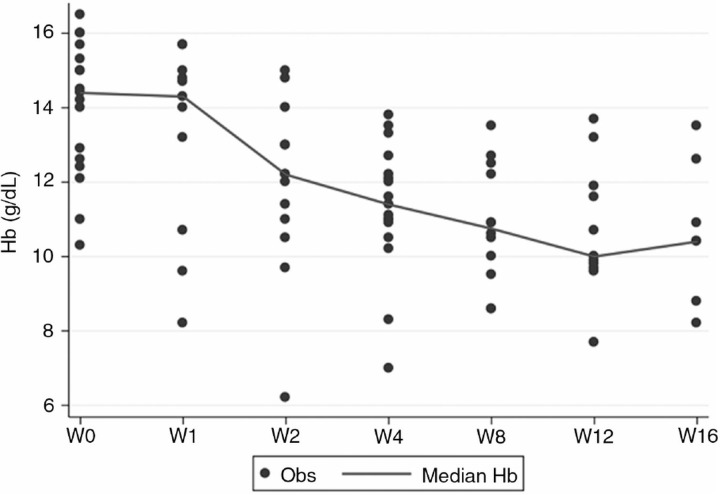
Serum creatinine levels and eGFR trends during follow-up period and hemoglobin trend during combination therapy.

**Table 1:** Multilevel regression analysis

Hb	β	[95% Conf. Interval]	P value
Weeks of follow-up	−0.61	−0.78; −0.45	<0.001
Creatinine (mg/dL)	−1.98	−3.63; −0.32	0.019
Male sex	1.86	0.37; 3.35	0.015
Age	−0.01	−0.23; 0.04	0.156
BMI	−0.05	−0.23; 0.14	0.641
Tenofovir Disoproxil Fumarate exposure	0.31	−0.34; 0.78	0.380
Proteinuria at baseline	0.44	−0.67; 0.86	0.476

